# The effects of grape seed extract supplementation on cardiovascular risk factors, liver enzymes and hepatic steatosis in patients with non-alcoholic fatty liver disease: a randomised, double-blind, placebo-controlled study

**DOI:** 10.1186/s12906-024-04477-3

**Published:** 2024-05-16

**Authors:** Parisa Ghanbari, Davoud Raiesi, Roghayeh Alboebadi, Ahmad Zarejavid, Mostafa Dianati, Hamidreza Razmi, Hadi Bazyar

**Affiliations:** 1 Student Research Committee, Ahvaz Jundishapur University of Medical Sciences, Ahvaz, Iran; 2 Department of Internal Medicine, School of Medicine, Ahvaz Jundishapur University of Medical Sciences, Ahvaz, Iran; 3 Nutrition and Metabolic Diseases Research Center, Clinical Sciences Research Institute, Ahvaz Jundishapur University of Medical Sciences, Ahvaz, Iran; 4 School of Medical and Life Sciences, Sunway University, Sunway City, Malaysia; 5 Department of Complex Genetics and Epidemiology, School of Nutrition and Translational Research in Metabolism, Maastricht University, Maastricht, Netherlands; 6 Department of Public Health, Sirjan School of Medical Sciences, Sirjan, Iran; 7 Student Research Committee, Sirjan School of Medical Sciences, Sirjan, Iran

**Keywords:** Grape seed extract, Non-alcoholic fatty liver disease, Hyperlipidemia, Hyperglycemia, Hypertension

## Abstract

**Background:**

Despite the high antioxidant potential of grape seed extract (GSE), very limited studies have investigated its effect on non-alcoholic fatty liver disease (NAFLD). Therefore, this study was conducted with the aim of investigating the effect of GSE on metabolic factors, blood pressure and steatosis severity in patients with NAFLD.

**Methods:**

In this double-blind randomized clinical trial study, 50 NAFLD patients were divided into two groups of 25 participants who were treated with 520 mg/day of GSE or the placebo group for 2 months. The parameters of glycemic, lipid profile, blood pressure and steatohepatitis were measured before and after the intervention.

**Results:**

The GSE group had an average age of 43.52 ± 8.12 years with 15 women and 10 men, while the placebo group had an average age of 44.88 ± 10.14 years with 11 women and 14 men. After 2 months of intervention with GSE, it was observed that insulin, HOMA-IR, TC, TG, LDL-c, ALT, AST, AST/ALT, SBP, DBP and MAP decreased and QUICKi and HDL-c increased significantly (*p*-value for all < 0.05). Also, before and after adjustment based on baseline, the average changes indicated that the levels of insulin, HOMA-IR, TC, TG, LDL-c, SBP, DBP, MAP in the GSE group decreased more than in the control group (p for all < 0.05). Furthermore, the changes in HDL-c were significantly higher in the GSE group (*p* < 0.05). The between-groups analysis showed a significant decrease in the HOMA-β and AST before and after adjustment based on baseline levels (*p* < 0.05). Moreover, the changes in QUICKi after adjustment based on baseline levels were higher in the GSE group than in the control group. Also, between-groups analysis showed that the severity of hepatic steatosis was reduced in the intervention group compared to the placebo group (*P* = 0.002).

**Conclusions:**

It seems that GSE can be considered one of the appropriate strategies for controlling insulin resistance, hyperlipidemia, hypertension and hepatic steatosis in NAFLD patients.

**Trial Registration:**

The clinical trial was registered in the Iranian Clinical Trial Registration Center (IRCT20190731044392N1). https://irct.behdasht.gov.ir/trial/61413. (The registration date: 30/03/2022)

## Introduction

Hepatosteatosis is defined as fat accumulation in the liver, comprising more than 5–10% of liver weight. Non-alcoholic fatty liver disease (NAFLD) is a term used to describe fatty liver disease that does not involve alcohol consumption. NAFLD encompasses a spectrum from benign steatosis to nonalcoholic steatohepatitis with inflammation (NASH) and liver cirrhosis [1]. Notably, NAFLD has significantly increased liver-related mortality, ranking as the 12th most common cause of death worldwide [2]. The global prevalence of NAFLD is reported to be 32.4%, and in Iran, this number reaches 40.8% [3]. Also, projections indicate that NAFLD will become the leading cause of death in Asia by 2030 [4].

The prevailing hypothesis regarding NAFLD pathogenesis is the multiple-hit hypothesis. According to this hypothesis, the initial trigger for NAFLD development is insulin resistance. Simultaneously, serum free fatty acids accumulate and transfer to hepatocytes, leading to increased hepatic lipogenesis from beta oxidation, ultimately resulting in steatosis [5]. Additionally, NAFLD disrupts vascular endothelium and serves as a primary marker of atherosclerosis. It is suggested that patients with fatty liver exhibit reduced vascular vasodilation compared to control subjects without steatosis [6]. NAFLD is considered a hepatic manifestation of metabolic syndrome, and its treatment primarily targets this syndrome. Conversely, the beneficial effects of polyphenols on metabolic syndrome have been demonstrated in numerous studies [7, 8].

Grape seed extract (GSE) is a food source rich in antioxidant potential, containing various polyphenols, including proanthocyanidins. Studies have reported that GSE significantly affects metabolic factors [9, 10]. Research has demonstrated that GSE’s anti-metabolic syndrome effects result from increased expression and concentration of adiponectin, inhibition of adipogenesis, and stimulation of lipolysis [10, 11]. Animal studies have confirmed that GSE treatment reduces fasting blood sugar (FBS), serum insulin, and HbA1c in diabetic rats. Furthermore, the positive effects of GSE on systolic blood pressure (SBP) are associated with increased production of nitric oxide and reduced expression of endothelin-1 [7].

Additionally, GSE has been shown to reduce the production of reactive oxygen species and inflammatory markers, as well as protect the liver against high-fat diets [12]. Numerous studies have investigated the effect of GSE on cardiovascular risk factors, and thus far, two studies have been conducted on NAFLD patients [13, 14]. However, none of the studies conducted on NAFLD patients have investigated insulin levels, homeostasis model assessment (HOMA-IR), homeostasis model assessment of beta-cell function (HOMA-β), quantitative insulin sensitivity check (QUICKi) index, atherogenic index of plasma (AIP), or blood pressure.

Therefore, the hypothesis of the present study was that GSE supplementation may play a beneficial role in improving NAFLD, as opposed to having no effect. Due to the confirmed beneficial effects of GSE on components of metabolic syndrome and the lack of sufficient randomized clinical trials (RCTs) evaluating the effects of GSE on metabolic factors in NAFLD patients, this study aimed to determine the effects of GSE supplementation on glycemic status, lipid profile, AIP, blood pressure, liver enzymes, and hepatic steatosis.

## Materials and methods

### Study design and population

This interventional study adhered to the ethical guidelines outlined in the 1975 Declaration of Helsinki and received approval from the Ethics Committee of Ahvaz Jundishapur University of Medical Sciences (Ethical Code: IR.AJUMS.REC.1400.704). The study was also registered with the Iran Clinical Trials Registry (registration code: IRCT20190731044392N1). Written informed consent was obtained from all patients at the study’s initiation.

**Fig. 1:**
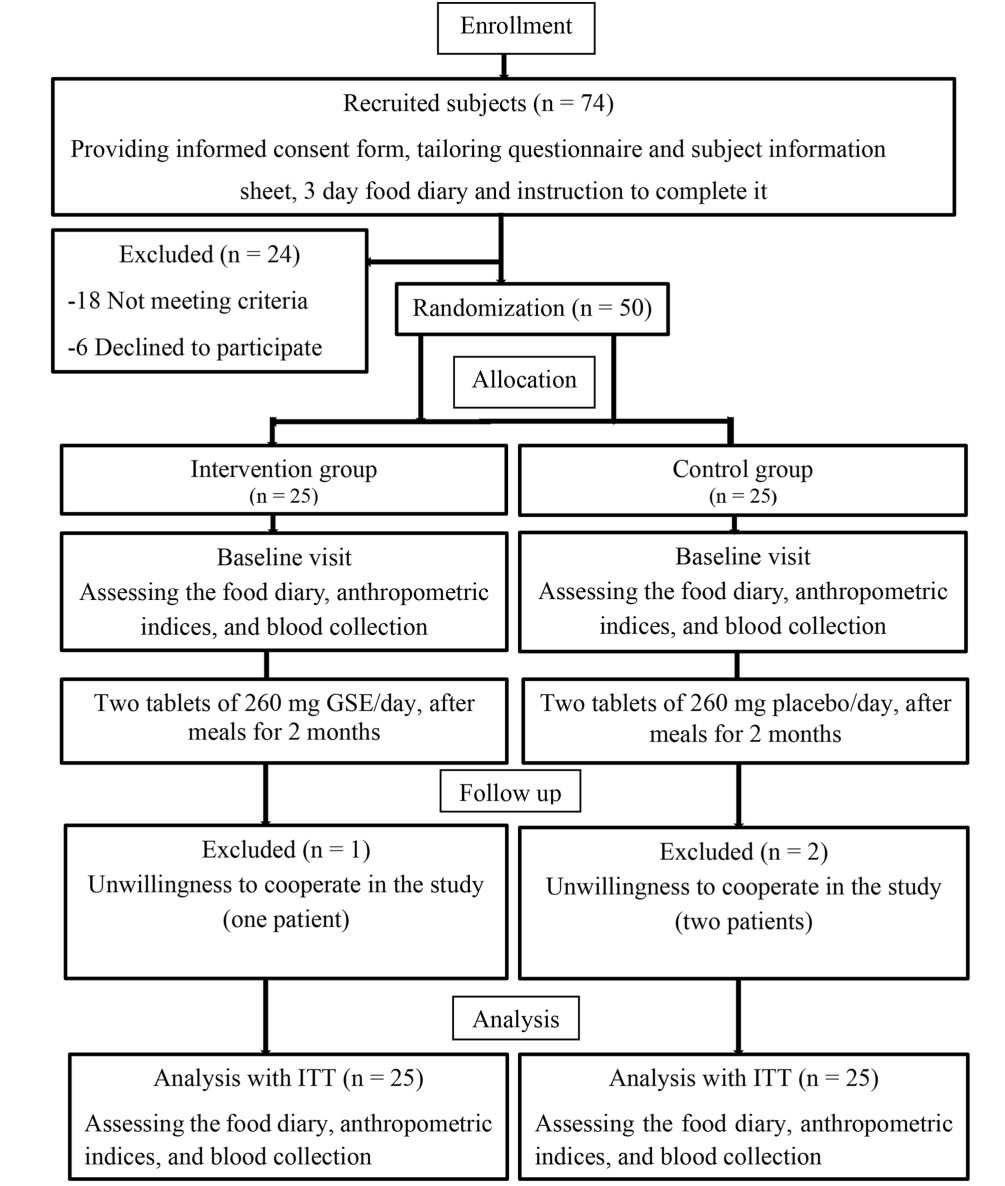
Stages of clinical trial progress

We followed the CONSORT standard flow diagram (Fig. 1) to conduct this randomized controlled trial. In this double-blind randomized controlled trial study, fifty patients with NAFLD were selected from those referred to the clinic of Shohadaye Hindijan Hospital between April and December 2022. The severity of hepatic steatosis in these patients was determined by a radiologist who was unaware of the study groups, using 3 to 5 MHz ultrasound. Hepatic steatosis was categorized into four groups based on liver echogenicity: Absent (score 0) (representing the normal condition), Mild (score 1) (characterized by echogenicity similar to the renal cortex), Moderate (score 2) (indicating a visible portal vein), and Severe (score 3) (associated with an invisible portal vein) [15, 16].

The inclusion criteria comprised moderate to severe steatosis detected via ultrasonography at the trial’s outset, age between 20 and 60 years, BMI between 25 and 35 kg/m², and patient willingness to participate in the study. Exclusion criteria included pregnancy and lactation, smoking, use of other food supplements, probiotics, or anti-inflammatory drugs, immunosuppressive drug use, and the presence of chronic diseases such as chronic liver disease, diabetes, kidney failure, thyroid disorders, and anemia, as well as adherence to special diets.

### Randomization and blinding

In this study, 50 patients were randomly divided into two supplement groups (*n* = 25) and a placebo group (*n* = 25) by assigning three-digit codes using Random Allocation Software (RAS) (Fig. 1). Allocation concealment was used for concealment. This work was done using opaque envelopes sealed with a random sequence (sequentially numbered, sealed, opaque envelopes). In this method, each of the generated random sequences (3-digit codes) was recorded on a card, and the cards were placed in the envelopes in order. To maintain a random sequence, the outer surface of the envelopes was numbered in the same order. Finally, the lid of the letter envelopes was glued, and they were placed in a box. The 3-digit codes were also mentioned on the supplement and placebo cans. At the time of participant registration, based on the order of arrival of eligible participants, the envelopes were opened in order, revealing the allocated group for each participant. In fact, each patient received a can containing a 3-digit code according to the code registered on the envelope, and the patients were placed in their respective groups. Coding was performed by a researcher outside the study. Both the researcher and patients were blinded during the study. Additionally, the type of intervention remained blind for the person performing the laboratory tests.

### Intervention

The patients in the supplement group received two tablets containing 260 mg of GSE daily (made by the Shari company, Iran) during their morning and evening snacks. Meanwhile, the patients in the control group received two 260 mg placebo tablets daily (produced by the Faculty of Pharmacy, Ahvaz Jundishapur University of Medical Sciences, Iran). These placebo tablets contained cellulose, silicon dioxide, magnesium stearate, and starch, and were similar in terms of shape, color, taste, and size to the supplement tablets. The duration of the study and the prescribed dose of GSE were determined based on previous studies [13, 17]. Patients were instructed at the start of the study to adhere to their regular diet and exercise routine. Additionally, patients were reminded to take supplements or placebo. Patient compliance was evaluated by counting the tablets after 2 months. Subjects who consumed less than 10% of the tablets were excluded. Possible side effects of GSE were evaluated during the study.

### Assessment of anthropometric indices

At the beginning of the study, the weight of each patient was measured with light clothes and with an accuracy of 100 g using a Seca scale (Germany), and the height of each patient was measured with a Seca height meter in a standing position without shoes using a height meter with an accuracy of 0.5 cm. Furthermore, waist circumference (WC) was measured with a tape measure with an accuracy of 0.5 cm while standing and in the narrowest part and in the area between the last rib and the iliac crest. Hip circumference (HC) was also measured while standing and at the widest circumference of the hip. The body mass index (BMI) of patients was calculated using the formula (dividing weight (kg) by height squared (m²)). To estimate WHR, WC was divided by HC. All measurements were performed by a trained expert.

### Biochemical evaluation

After fasting for 12 h, blood was collected and centrifuged at 2500 rpm for 10 min. The serum samples were frozen immediately at -80 °C until analyzed. The levels of liver enzymes (AST and ALT) were measured by colorimetric method using Parsazmun kits (Tehran, Iran). Total cholesterol (TC), triglyceride (TG), and high-density lipoprotein cholesterol (HDL-c) levels were evaluated using enzymatic kits (Pars Azmun, Iran). To calculate low-density lipoprotein cholesterol (LDL-C), the Friedewald formula was used. FBS level was assessed via the enzymatic colorimetric method using glucose oxidase, and insulin levels were measured by an insulin ELISA kit (Monobind, USA). HbA1c was measured by the HPLC method with a Ds5 device. The homeostasis model assessment [HOMA-IR], Homeostasis model assessment of beta-cell function (HOMA-β), and quantitative insulin sensitivity check (QUICKi) index were calculated according to the following formula: HOMA-IR = [FBS (mmol/L) fasting insulin (µIU/mL)]/22.5, QUICKi = 1/[log (I0) + log (G0)], and HOMA- β = [360 fasting insulin (µIU/mL)]/[FBS (mg/dL)63]. Furthermore, the atherogenic index of plasma (AIP) was calculated as log (TG/HDL-c) [18]. These measurements were obtained at pre-intervention and after 2 months of monitoring.

### Assessment of physical activity, food intake and blood pressure

At the beginning of the study and at the end of eight weeks, a 3-day food record (two non-holiday days and 1 holiday day) and physical activity intensity according to Metabolic Equivalents (MET) were assessed using a physical activity questionnaire (IPAQ) [19]. The information obtained through the 3-day food record questionnaire was analyzed using Nutritionist 4 (Nut 4) software. SBP and diastolic blood pressure (DBP) were measured using an automatic oscillometric device on the right arm after five minutes of rest in a sitting position with an accuracy of 2 mmHg, both at the beginning and end of the study. Blood pressure was measured twice, and its average was recorded. The pulse pressure (PP) and mean arterial pressure (MAP) were computed using the following formula: PP = SBP-DBP, MAP= (SBP + 2DBP)/3 [20].

### Sample size

The sample size was determined based on insulin levels as the main result. According to a study by Taghizadeh et al., study [21], with α = 0.05, 90% power, and assuming a probable 25% dropout rate, the sample size was estimated to be 25 participants per group. The sample size formula considering σ1 = 25.2, σ2 = 23.4, µ1 = 1.8, µ2 = -23.4 is presented below.

($$n=\frac{{\left({z}_{1}- \frac{\alpha }{2}+ {z}_{1}-\beta \right)}^{2}\left({{\delta }_{1}}^{2}+{{\delta }_{2}}^{2}\right)}{{({\mu }_{1}- {\mu }_{2})}^{2}}$$)

### Statistical analysis

The normality of data distribution was evaluated by the Kolmogorov-Smirnov test. Paired sample t-test or Wilcoxon test were used to compare intragroup changes in variables, and the comparison between the studied variables of the two GSE and placebo groups was done using the independent sample t-test. Analysis of covariance (ANCOVA) was used to evaluate the variables between two groups after adjusting for baseline. All values ​​were reported based on the mean ± standard deviation. Based on the dropout rate of participants, the intention-to-treat (ITT) method was utilized. Missing values were imputed by calculating the median of the available data for each variable with missing values. Data analysis was done using SPSS23 software. *P* < 0.05 was considered statistically significant.

## Results

In the present study, 74 patients with NAFLD were investigated, of which 50 patients met the conditions for inclusion in the study. Finally, 50 patients with NAFLD were divided into one of the two groups: GSE or placebo. During the intervention, one participant in the intervention group with GSE and two in the placebo group were excluded from the study due to lack of interest in continuing the study. Nevertheless, after using the ITT method, at the end, 25 people in each group were subjected to statistical analysis. No side effects were reported in the patients during the consumption of GSE supplements or placebo.

### Basic characteristics

All data showed normal distributions. The mean age of the participants in the GSE group was 43.52 ± 8.12 years, consisting of 15 women and 10 men. In comparison, the placebo group had a mean age of 44.88 ± 10.14 years, with 11 women and 14 men. Average anthropometric indexes, age, sex, and the severity of liver steatosis did not differ significantly at the beginning (Table 1) (*P* ≥ 0.05). According to the BMI patients, both groups were in the first-degree obesity category (31.80 ± 5.17 in the placebo group and 32.35 ± 4.34 in the GSE group). Also, race, education, occupation and medications (data not shown) were examined, and no significant difference was observed between the two groups (*P* ≥ 0.05). The intake of energy and macronutrients (shown in Table 2) had no statistically significant differences in the two groups (*P* ≥ 0.05).

**Table 1:** The characteristics of subjects at baseline

Variables	Control group (*n* = 25)	Intervention group (*n* = 25)	^*^P-value
**Gender** (n) (%)			
Female	11 (44)	15 (68)	
Male	14 (56)	10 (32)	0.25 ^a^
**Age (years)**	44.88 ± 10.14	43.52 ± 8.12	0.60
**Height (cm)**	168.08 ± 9.77	166.12 ± 8.14	0.44
**Weight (kg)**	87.72 ± 5.77	87.48 ± 5.77	0.84
**BMI (kg/m**^**2**^)	31.42 ± 3.63	31.52 ± 3.58	0.87
**WC (cm)**	108.16 ± 9.21	110.72 ± 6.38	0.25
**HC (cm)**	114.72 ± 6.74	117.08 ± 7.84	0.40
**Race (n) (%)**			
Fars	19 (76)	21 (84)	0.75 ^a^
Lor	2 (8)	1 (4)	
Arab	4 (16)	3 (12)	
**Education (n) (%)**			
Illiterate – elementary	6 (24)	1 (4)	0.13 ^a^
Middle – school	8 (32)	13 (52)	
High – school	3 (12)	5 (20)	
College	8 (32)	6 (24)	
**Job (n) (%)**			
Unemployed			
Labor	9 (36)	8 (32)	0.92 ^a^
Housekeeper	11 (44)	11 (44)	
Employee	5 (20)	6 (24)	
**Physical Activity (met-min/week)**	354.40 ± 169.48	334.00 ± 130.10	0.63

BMI, body mass index; WC, waist circumference; HC, hip circumference

Values are expressed as means ± SD. *P* < 0.05 was considered as significant

*P < 0.05 was considered as significant using Independent t-test between the two groups at baseline

^*a*^*P* < 0.05 was considered as significant using Chi-square test

**Table 2:** Mean ± SD of energy, macronutrients, and micronutrients intake at baseline and post- intervention

Variables	Baseline (*n* = 25)	Post-intervention (*n* = 25)	*P*-value**
**Energy (kcal/d)**			
Control group	1995.51 ± 161.86	2021.09 ± 139.25	0.35
Intervention group	2061.07 ± 172.90	2052.34 ± 164.97	0.64
* P-value* *	0.17	0.72	
**Carbohydrate (g/d)**			
Control group	256.79 ± 20.86	256.64 ± 17.91	0.96
Intervention group	263.83 ± 22.38	260.07 ± 22.22	0.14
* P-value* *	0.25	0.60	
**Protein (g/d)**			
Control group	78.60 ± 7.02	79.48 ± 5.69	0.43
Intervention group	81.56 ± 6.43	80.45 ± 6.28	0.15
* P-value* *	0.12	0.57	
**Fat (g/d)**			
Control group	70.84 ± 5.91	71.14 ± 5.20	0.74
Intervention group	72.65 ± 5.45	71.90 ± 5.70	0.23
* P-value* *	0.26	0.49	
**Cholesterol (g/d)**			
Control group	167.11 ± 26.13	163.41 ± 21.60	0.73
Intervention group	136.28 ± 24.17	125.81 ± 34.33	0.81
* P-value* *			
**Vitamin A (mcg/d)**			
Control group	369.14 ± 115.89	371.28 ± 74.57	0.64
Intervention group	385.77 ± 101.64	383.97 ± 98.43	0.75
* P-value* *	0.19	0.35	
**Beta-Carotene (mcg/d)**			
Control group	4264.71 ± 1631.69	4250.41 ± 1132.41	0.89
Intervention group	4572.55 ± 1738.14	4157.74 ± 1147.87	0.37
* P-value* *	0.44	0.26	
**Selenium (mcg /d)**			
Control group	48.78 ± 19.38	51.11 ± 14.49	0.81
Intervention group	56.34 ± 18.09	51.73 ± 22.71	0.29
* P-value* *	0.16	0.90	
**Vitamin C (mg/d)**			
Control group	100.05 ± 36.01	95.58 ± 27.62	0.18
Intervention group	89.76 ± 30.45	92.48 ± 29.92	0.27
* P-value* *	0.28	0.45	
**α-tocopherol (mg/d)**			
Control group	7.25 ± 1.70	7.37 ± 1.41	0.29
Intervention group	6.70 ± 1.90	7.55 ± 2.21	0.12
* P-value* *	0.16	0.74	
**Vitamin E (mg/d)**			
Control group	2.32 ± 0.67	2.27 ± 0.55	0.11
Intervention group	2.26 ± 0.61	2.25 ± 0.52	0.16
* P-value* *	0.73	0.55	

*P < 0.05 was considered as significant at baseline and significant post-intervention using Independent T-test between two groups

**P < 0.05 was considered as significant using Paired T-test

### GSE and glycemic parameters

At baseline, there was no significant difference between the mean serum concentrations of the glycemic parameters (*P* ≥ 0.05). As shown in Tables 2 and 3 months of supplementation with GSE had no significant effect on FBS and HbA1c levels (*P* ≥ 0.05), while the average insulin levels and HOMA-IR and QUICKi in the intervention group with GSE improved significantly (14.26 ± 3.26 vs. 12.29 ± 2.26, *p* = 0.01; 3.35 ± 0.88 vs. 2.83 ± 0.60, *p* = 0.01; 0.32 ± 0.01 vs. 0.33 ± 0.01, *p* = 0.002). Also, the between-group analysis showed that the average changes in insulin serum levels and HOMA-IR and HOMA-β indices in the intervention group with GSE were significantly lower than the control group (0.97 ± 0.52 vs. 0.90 ± 0.03, *p* = 0.04; 57.80 ± 21.16 vs. 53.97 ± 11.14, *p* = 0.04). After adjusting the data based on baseline levels, it was observed that the significance of insulin and HOMA-IR increased (*p* = 0.002, *p* < 0.001 respectively), but HOMA-β was not statistically significance. We also found that the QUICKi index became statistically significant after adjustment for baseline levels (*p* = 0.001).

### GSE and lipid profile

At baseline, the lipid profiles (TG, TC, LDL-c, HDL-c, and VLDL) of the intervention and control groups did not differ significantly (*P* ≥ 0.05). The paired sample t-test analysis showed that the average levels of TC, TG, LDL-c, and LDL/HDL ratio in the GSE group decreased significantly (191.14 ± 23.70 vs. 182.58 ± 21.59, *p* < 0.001; 158.84 ± 34.26 vs. 173.24 34.26 ± 34.36 vs. 158.84 ± 34.26, *p* < 0.001; 23.21 ± 113.21 vs. 101.89 ± 22.88; *p* < 0.001; 0.67 ± 2.69 vs. 2.13 ± 0.58, *p* < 0.001, respectively) and the average HDL-c level increased (43.28 ± 8.34 vs. 48.91 ± 7.32, *p* < 0.001). Also, we found that the average AIP index in the intervention group improved significantly (0.15 ± 0.60 vs. 0.14 ± 0.50, *p* = 0.01).

Between groups analysis also showed that the average changes of TC, TG, and LDL-c in the intervention group with GSE had a significant difference from the control group (− 8.56 ± 9.44 vs. 6.54 ± 21.58, *p* = 0.002; -14.10 ± 16.16 vs. 1.32 ± 32.36, *p* = 0.002, *p* = 0.03; − 11.31 ± 10.53 vs. 5.22 ± 22.55, *p* = 0.002). Also, the average HDL-c changes were significantly higher than the control group (5.63 ± 7.32 vs. 1.05 ± 4.70, *p* = 0.004). Investigating the average changes of AIP and LDL/HDL ratio also showed that they were significantly lower in the intervention group (-0.09 ± 0.06 vs. -0.008 ± 0.09, *p* < 0.001; -0.56 ± 0.46 vs. 0.07 ± 0.62, *p* < 0.001). After adjusting the results based on the baseline levels, no significant difference was found and it was observed that the difference in the changes of TG, TC, LDL-c, HDL-c, LDL, HDL and AIP levels between the two groups was significant (*P* < 0.001, *p* = 0.01, *p* < 0.001, *p* < 0.001, *p* < 0.001, *p* < 0.001) (Table 4).

**Table 3:** Glycemic status at baseline and post-intervention

Variables	Intervention group (*n* = 25)	Control group (*n* = 25)	*P*-Value^**^	*P*-Value***	*P*-Value****
**FBS (mg/dl(**					
Baseline	95.12 ± 10.23	99.48 ± 6.80	0.08		
After 2 months	93.09 ± 7.11	98.07 ± 7.04	0.01		
P-Value*	0.12	0.18			
Difference	-2.02 ± 6.36	-1.40 ± 5.13		0.70	0.08
**HbA1c (%)**					
Baseline	5.76 ± 0.57	5.95 ± 0.53	0.21		
After 2 months	5.71 ± 0.51	5.91 ± 0.51	0.16		
* P-Value* ^*^	0.27	0.12			
Difference	-0.04 ± 0.21	-0.04 ± 0.12		0.87	0.49
**Insulin(µIU/mL)**					
Baseline	14.26 ± 3.26	13.79 ± 2.74	0.58		
After 2 months	12.29 ± 2.26	14.28 ± 2.27	0.003		
* P*-Value^*^	0.01	0.50			
Difference	-1.96 ± 3.88	0.49 ± 3.63		0.02	0.002
**HOMO-IR**					
Baseline	3.35 ± 0.88	3.37 ± 0.68	0.91		
After 2 months	2.83 ± 0.60	3.41 ± 0.53	0.001		
* P-Value* ^*^	0.01	0.84			
Difference	-0.52 ± 0.97	0.03 ± 0.90		0.04	< 0.001
**HOMA-β**					
Baseline	176.85 ± 67.38	149.54 ± 44.41	0.09		
After 2 months	155.69 ± 48.52	160.69 ± 48.52	0.002		
* P-Value* ^*^	0.08	0.31			
Difference	-21.16 ± 57.80	11.14 ± 53.97		0.04	0.213
**QUICKi**					
Baseline	0.32 ± 0.01	0.31 ± 0.01	0.31		
After 2 months	0.33 ± 0.01	0.32 ± 0.01	0.71		
* P-Value* ^*^	0.002	0.51			
Difference	0.009 ± 0.01	0.002 ± 0.01		0.14	0.001

FBS, Fasting Blood Sugar; HbA1c, Glycated Hemoglobin; HOMA-IR, Homeostatic Model Assessment for Insulin Resistance; HOMA-β, Homeostatic Model Assessment for Beta Cell Function; QUICKi, Quantitative Insulin Sensitivity Check Index. Values are expressed as means ± SD. *P < 0.05 was considered as significant using Paired t-test. **P < 0.05 was considered as significant using Independent t-test between the two groups at baseline and post-intervention. ***P < 0.05 was considered as significant difference using Independent t-test between the two groups post-intervention. ****P < 0.05 was considered as significant using Analysis of covariance (ANCOVA) between the two groups post-intervention after adjusting for baseline

**Table 4:** Lipid profile and AIP and at baseline and post-intervention

Variables	Intervention group (*n* = 25)	Control group (*n* = 25)	*P*-Value^**^	*P*-Value***	*P*-Value****
**LDL-c )mg/dl)**					
Baseline	113.21 ± 23.21	120.60 ± 29.27	0.32		
After 2 months	101.89 ± 22.88	125.82 ± 30.15	0.003		
* P*-Value*	< 0.001	0.74			
Difference	− 11.31 ± 10.53	5.22 ± 22.55		0.002	< 0.001
**TG (mg/dl)**					
Baseline	173.24 ± 34.36	175.80 ± 37.24	0.80		
After 2 months	158.84 ± 34.26	177.12 ± 38.70	0.08		
* P*-Value^*^	< 0.001	0.84			
Difference	-14.10 ± 16.16	1.32 ± 32.36		0.03	0.01
**TC )mg/dl)**					
Baseline	191.14 ± 23.70	197.64 ± 33.99	0.43		
After 2 months	182.58 ± 21.59	204.18 ± 33.67	0.01		
* P*-Value^*^	< 0.001	0.14			
Difference	− 8.56 ± 9.44	6.54 ± 21.58		0.002	< 0.001
**VLDL (mg/dl)**					
Baseline	44.53 ± 19.52	43.24 ± 13.66	0.78		
After 2 months	45.09 ± 15.95	46.13 ± 12.96	0.80		
* P*-Value^*^	0.89	0.26			
Difference	0.55 ± 12.76	2.89 ± 12.69		0.52	0.54
**HDL-c (mg/dl)**					
Baseline	43.28 ± 8.34	41.88 ± 7.42	0.53		
After 2 months	48.91 ± 7.32	42.93 ± 7.33	0.006		
* P*-Value^*^	< 0.001	0.27			
Difference	5.63 ± 7.32	1.05 ± 4.70		0.004	< 0.001
**LDL/HDL**					
Baseline	2.69 ± 0.67	2.95 ± 0.76	0.21		
After 2 months	2.13 ± 0.58	3.02 ± 0.87	< 0.001		
* P*-Value^*^	< 0.001	0.57			
Difference	-0.56 ± 0.46	0.07 ± 0.62		< 0.001	< 0.001
**AIP**					
Baseline	0.60 ± 0.15	0.61 ± 0.13	0.65		
After 2 months	0.50 ± 0.14	0.61 ± 0.12	0.008		
* P*-Value^*^	0.01	0.67			
Difference	− 0.09 ± 0.06	-0.008 ± 0.09		< 0.001	< 0.001

LDL-c, Low-Density Lipoprotein Cholesterol; TG, Triglycerides; TC, Total Cholesterol; VLDL, Very Low-Density Lipoprotein; HDL-c, High-Density Lipoprotein Cholesterol; AIP, Atherogenic Index of Plasma. Values are expressed as means ± SD. *P < 0.05 was considered as significant using Paired t-test. **P < 0.05 was considered as significant using Independent t-test between the two groups at baseline and post-intervention. ***P < 0.05 was considered as significant difference using Independent t-test between the two groups post-intervention. ****P < 0.05 was considered as significant using Analysis of covariance ± ANCOVA) between the two groups post-intervention after adjusting for baseline

### GSE and blood pressure

At the beginning of the study, the baseline blood pressure of the patients in the two groups was not significantly different (*p* > 0.05). However, after 2-month of intervention with GSE, we observe that the mean arterial pressure (MAP), SBP and DBP in the intervention improved significantly (108.40 ± 11.06 vs. 95.60 ± 7.37, *p* < 0.001; 132.80 ± 13.39 vs. 118.00 ± 9.12, *p* < 0.001; 97.60 ± 9.25 vs. 84.40 ± 8.20, *p* < 0.001) while these results were not seen in the control group. On the other hand, the between-group results also showed that the average changes in MAP, SBP and DBP were significantly lower than the control group (-13.73 ± 7.09 vs. 0.40 ± 7.02, *p* < 0.001; 8.22 ± 14.80 vs. -9.78 ± 0.40, *p* < 0.001; -14.40 ± 7.94 vs. 6.72 ± 0.80, *p* < 0.001). After adjusting the results, it was observed that the changes in the average MAP, SBP and DBP between the two groups were significant (p for all < 0.001) (Table 5).

### GSE and liver enzymes and severity of hepatic steatosis

We found that the levels of ALT and AST decreased after 2-month of supplementation with GSE (34.87 ± 8.70 vs. 24.28 ± 8.61, *p* < 0.001; 21.40 ± 3.80 vs. 18.75 ± 4.08, *p* = 0.02). Moreover, the mean levels of AST/ALT significantly rose following supplementation. The results of the independent sample t-test between groups also showed significant changes in AST/ALT and ALT in the intervention group compared to the control group (-10.59 ± 4.85 vs. -1.26 ± 7.04, *p* < 0.001), while this significant difference was not seen in the AST enzyme (*p* > 0.05). However, after adjusting the AST changes based on its baseline level, a significant difference was observed between the two groups (*P* = 0.03) (Table 5).

According to the ultrasound, performed at the beginning of the study, none of the participants had normal hepatic steatosis. On the other hand, by comparing the baseline of hepatic steatosis between the intervention and placebo groups, no significant difference was seen between the two groups (*p* > 0.05). By performing the Wilcoxon test, it was observed that 60% of patients with moderate and severe steatosis reached normal or mild steatosis (*P* < 0.001). Also, at the end of the study, the chi-square test showed a significant difference in the severity of hepatic steatosis between the two groups (*P* = 0.002) (Table 5).

**Table 5:** Blood pressure markers, Liver enzymes and hepatic steatosis at baseline and post-intervention

Variables	Intervention group (*n* = 25)	Control group (*n* = 25)	*P*-Value^**^	*P*-Value***	*P*-Value****
**SBP (mmHg)**					
Baseline	132.80 ± 13.39	130.80 ± 12.22	0.55		
After 2 months	118.00 ± 9.12	130.40 ± 14.85	0.001		
* P*-Value^*^	< 0.001	0.84			
Difference	-14.80 ± 8.22	-0.40 ± 9.78		< 0.001	< 0.001
**DBP (mmHg)**					
Baseline	97.60 ± 9.25	96.60 ± 10.27	0.36		
After 2 months	84.40 ± 8.20	97.40 ± 10.71	< 0.001		
* P*-Value^*^	< 0.001	0.55			
Difference	-14.40 ± 7.94	0.80 ± 6.72		< 0.001	< 0.001
**PP (mmHg)**					
Baseline	35.20 ± 9.62	34.20 ± 8.12	0.39		
After 2 months	33.60 ± 9.07	33.00 ± 11.18	0.83		
* P*-Value^*^	0.38	0.43			
Difference	-1.60 ± 8.98	-1.20 ± 7.53		0.86	0.95
**MAP (mmHg)**					
Baseline	109.33 ± 9.81	108.00 ± 10.27	0.64		
After 2 months	95.60 ± 7.37	108.40 ± 11.06	< 0.001		
* P*-Value^*^	< 0.001	0.77			
Difference	-13.73 ± 7.09	0.40 ± 7.02		< 0.001	< 0.001
**ALT (mg/dl)**					
Baseline	34.87 ± 8.70	36.32 ± 8.58	0.55		
After 2 months	24.28 ± 8.61	35.06 ± 8.70	0.001		
* P*-Value^*^	< 0.001	0.37			
Difference	-10.59 ± 4.85	-1.26 ± 7.04		< 0.001	< 0.001
**AST (mg/dl)**					
Baseline	21.40 ± 3.80	22.28 ± 6.84	0.57		
After 2 months	18.75 ± 4.08	22.28 ± 6.84	0.03		
* P*-Value^*^	0.02	0.38			
Difference	-2.64 ± 5.55	-0.99 ± 5.60		0.30	0.03
**AST/ALT**					
Baseline	0.63 ± 0.10	0.62 ± 0.18	0.90		
After 2 months	0.88 ± 0.41	0.63 ± 0.15	0.007		
* P*-Value^*^	0.009	0.007			
Difference	0.25 ± 0.36	0.003 ± 0.21		0.006	0.002
**Steatosis n (%)**					
Baseline					
Normal	0 (0)	0 (0)			
Mild	0 (0)	0 (0)	0.20 ^b^		
Moderate	16 (64)	20 (80)			
Severe	9 [35]	5 [19]			
After 2 months					
Normal	1 [4]	0 (0)			
Mild	14 [55]	2 [7]	0.002 ^b^		
Moderate	9 [35]	19 (68)			
Severe	1 [4]	4 [19]			
* P*-Value ^a^	< 0.001	0.08			

SBP, Systolic Blood Pressure; DBP, Diastolic Blood Pressure; PP, Pulse Pressure; MAP, Mean Arterial Pressure; ALT, Alanine Aminotransferase; AST, Aspartate Aminotransferase. Values are expressed as means ± SD. *P < 0.05 was considered as significant using Paired t-test. **P < 0.05 was considered as significant using Independent t-test between the two groups at baseline and post-intervention. ***P < 0.05 was considered as significant difference using Independent t-test between the two groups post-intervention. ****P < 0.05 was considered as significant using Analysis of covariance ± ANCOVA) between the two groups post-intervention after adjusting for baseline. ^a^*P* < 0.05 was considered as significant using Wilcoxon test. ^b^*P* < 0.05 was considered as significant using Chi-square test

## Discussion

There have been limited studies of supplementing with GSE in NAFLD patients. As a result of this study, positive effects of GSE on average insulin level, insulin resistance, lipid profile, blood pressure and severity of hepatic steatosis were seen in patients with NAFLD. NAFLD is a disease associated with metabolic disorders in a vicious cycle [22]. Studies have shown that insulin resistance is one of the main causes of NAFLD [23], and that the oxidative stress created in NAFLD can play a role in increasing insulin resistance, lipid disorders [24] and hypertension [25]. The association of GSE with metabolic disorders has been reported in various studies. Controlling each of the metabolic variables can play an important role in preventing NAFLD.

### GSE and glycemic parameters

Dysfunction of insulin causes damage to the pathway of carbohydrates and fats, which provides the basis for the flow of fatty acids to the liver, increases the synthesis and storage of triglycerides, and finally increases the levels of inflammation and steatosis in the body [23]. Therefore, the control of insulin resistance can be considered one of the important solutions to improving NAFLD. As a result of the present study, we observed a decrease in average insulin levels and an improvement in HOMA-IR, HOMA-β, and QUICKi with GSE supplementation. In line with the results of our study, human studies have also observed the improvement of average insulin levels and insulin resistance index (HOMA-IR or HOMA-β) in the group supplemented with GSE [21, 26, 27]. Our investigation observed a mean difference in HOMA-IR of -0.52 ± 0.97 post-intervention. The minimal clinically important difference (MCID) for HOMA-IR has been reported to vary across different studies, with values ranging from 0.26 [28] to specific cut-off values like 3.63 [29] for identifying diabetes mellitus. This variability underscores the importance of context-specific considerations when interpreting changes in HOMA-IR levels. While the observed mean difference falls below the commonly reported MCID thresholds, the clinical relevance of this change warrants further exploration, especially in the context of our study population.

In addition, in the animal study conducted by Bao et al., a decrease in insulin levels, FBS, and HbA1c was seen in diabetic rats treated with GSE [7]. The lack of effect of GSE on FBS and HbA1c in the present study could be attributed to the normal levels of HbA1c variables and FBS in the baseline state. Different mechanisms for the effect of GSE on glycemic parameters have been reported in the previous studies. It has been said that the phenols in GSE can increase the expression of proteins related to the insulin signaling pathway and the expression of glycogen synthase mRNA [9]. On the other hand, the proanthocyanidins present in the GSE can be effective in improving the glycemic profile by increasing pancreatic glutathione and decreasing lipid peroxidation as well as total nitrate/nitrite levels in the pancreas [30]. According to the unadjusted and adjusted analysis, there was a significant decrease in insulin and HOMA-IR between the two groups. However, HOMA-β and QUICKi showed significant differences between groups before and after adjustment. Contrary to the findings, in a study conducted on patients with metabolic syndrome for 4 weeks, there was no significant difference in HOMA-IR and average insulin in the intervention group with freeze-dried grape powder compared to the control group [31]. The difference in the duration of the study and the type of intervention may have been effective factors on the results. Almost similar findings were observed in studies demonstrating an improvement in the effects of GSE on insulin levels and insulin resistance. It has been shown that GSE can be effective in improving insulin secretion by inhibiting dipeptidyl peptidase 4 in the intestine and increasing the activity of GLP-1 [32].

### GSE and lipid profile

It was seen in the present study that taking 520 mg/day of GSE is effective in improving TC, TG, LDL-c and HDL-c levels. In addition, AIP was used to show the predictive capacity of lipid profiles for cardiovascular disease. The findings of this study showed that receiving 2 months of GSE can have positive results on the AIP index.

In various studies, the relationship between dyslipidemia and NAFLD has been reported. A cohort study conducted in Iran showed high levels of TC, LDL/HDL ratio and low HDL in patients with NAFLD [33]. Insulin resistance, by causing dyslipidemia, can lead to increased oxidative stress and lipid peroxidation, all of which contribute to NAFLD [34]. Yogalakshmi et al. suggested that grape seed proanthocyanidins can stimulate enzymes involved in β-oxidation of lipids by affecting the peroxisome proliferator-activated receptor α. PPARs are high-potential targets for NAFLD therapy [35]. In confirmation of these findings, human studies confirmed the hypoglycemic, hypolipidemic, and anti-atherogenic effects of GSE [13, 36]. However, in a study conducted on 40 female volleyball players, no significant difference was found in the lipid profile levels between the two intervention groups with GSE and the placebo group [37]. In one of the meta-analysis studies, the reduction of LDL-c and TG levels with the consumption of GSE has been reported, and on the other hand, it has been stated that receiving an amount of less than 300 mg of GSE for less than 10 weeks probably does not affect HDL-c and TC [38]. Therefore, the positive effects seen in the present study may be due to the higher dose of GSE prescribed in the study. Also, the type of intervention group or the basic levels of lipid profiles may be the reasons for the difference between the results of this study and other studies. The anti-hyperlipidemic effect of GSE suggests that the high antioxidant potential caused by proanthocyanidin can prevent cholesterol accumulation by scavenging free radicals and inhibiting LDL-c oxidation. It has been reported that GSE can lead to the inhibition of enzymes effective in the digestion of fats and prevent micellization of cholesterol by bile acid [39]. In the present study, before and after adjusting the results, the levels of LDL, HDL, TG, and TC showed a significant difference in the between-groups analysis. Contrary to these studies, Odai et al. showed that the consumption of 200 and 400 mg/day of GSE for 12 weeks does not create a significant difference in HDL and LDL levels between the intervention and control groups [17]. However, in another study conducted on obese or overweight adult individuals, lipid parameters and AIP were significantly improved [36].

The mean difference in LDL cholesterol of -11.31 ± 10.53 seen in our study surpasses the MCID reported for LDL cholesterol, which stands at 3.87 mg/dl [40]. Although the MCID for TC remains unspecified in our search results, the notable reduction in LDL cholesterol post-intervention accentuates the favorable impact of the treatment on lipid parameters. This marked decrease underscores a potential beneficial effect on cardiovascular risk, warranting attention in the management of dyslipidemia. Additionally, it has been shown that the MCID for TG varies depending on the study duration. At 6 months, a decrease of 0.30 mmol/L is considered a clinically meaningful benefit, surpassing the MCID threshold of 0.09 mmol/L [28]. Similarly, at 12 months, a decrease of 0.32 mmol/L is also deemed beneficial, exceeding the MCID of 0.09 mmol/L [28]. The mean difference of TG before and after the intervention in our study was observed to be -14.10 ± 16.16 mg/dl. This considerable reduction signifies a noteworthy positive impact on lipid profiles, highlighting the effectiveness of the GSE intervention in managing TG levels within the studied population. GSE can be effective in improving lipid profile levels by reducing acyl-coenzyme A, cholesterol acyltransferase and inhibiting microsomal triglyceride transfer protein, and increasing fatty acid oxidation [41].

### GSE and blood pressure

Another important finding is that GSE intervention leads to a decrease in MAP, SBP and DBP in patients with NAFLD. Studies have shown that the prevalence of hypertension in patients with NAFLD exceeds 40% [42]. It is suggested that inflammation in NAFLD can increase the iNOS / eNOS ratio, which leads to endothelial dysfunction, increased insulin resistance, and oxidative stress [25, 43]. Additionally, chronic inflammation has been observed to upregulate the renin-angiotensin system (RAS) in NAFLD [44].

Studies have reported that the primary mechanism of lowering blood pressure by GSE is due to the stimulation of nitric oxide release and its anti-inflammatory and antioxidant properties [45, 46]. Additionally, a humane investigation of healthy individuals conducted by Schön et al., revealed that a 4-month intervention with 300 mg GSE could successfully lower blood pressure. It was demonstrated that GSE is effective in reducing soluble intercellular adhesion molecule-1 (sICAM) and endothelin-1 secretion [47]. The sICAM and endothelin-1 are implicated in the activation or damage of cells such as platelets and endothelium and could be associated with high blood pressure [48]. Moreover, in a clinical trial conducted with doses of 200 mg and 400 mg of GSE in patients with high blood pressure, it was observed that MAP, DBP, and SBP were significantly lower with the administration of a higher dose of GSE than in the control group [17].

The alterations in blood pressure parameters post-intervention revealed compelling findings. A substantial decrease in SBP by -14.80 ± 8.22, DBP by -14.40 ± 7.94, and MAP by -13.73 ± 7.09 was noted. While the specific MCID for blood pressure in our search results remains unspecified, previous research suggests that even a modest reduction of 2 mmHg in SBP could translate to a significant decrease in cardiovascular mortality risk [49]. The substantial reductions observed in our study signal promising implications for cardiovascular health and underscore the potential clinical significance of the intervention on blood pressure management. The valuable effects of the intake of GSE on blood pressure could be explained by its decrease of the cell damage caused by free radicals and improved endothelial function through an increase in enhancing nitric oxide bioactivity [50]. Similarly, Mas-Capdevila et al., reported that GSE can be effective on blood pressure by downregulating the expression of the main endothelial vasoconstrictor, ET-1, and inducing the upregulation of the NO-enhancer Sirt-1 mRNA [51]. However, despite these findings, no significant effect of GSE on SBP and DBP was seen in some studies [17, 52] which might be the reason for the difference between the results of these studies and the present study, the duration of the intervention, the difference in doses used in studies or the sample size. It has also been shown that GSE can be effective in reducing blood pressure with its anti-obesity effects in addition to reducing cardiac output [53].

### GSE and liver enzymes and severity of hepatic steatosis

In addition to the above-mentioned variables, this study evaluated the severity of hepatic steatosis using ultrasound. The findings showed that receiving the GSE supplement at a rate of 520 mg per day for two months could effectively reduce the severity of hepatic steatosis and improve liver enzyme levels. In line with this study, human and animal studies have revealed a significant decrease in liver enzymes and the severity of liver steatosis after the intake of GSE [13, 54]. Considering the high antioxidant potential of GSE and the role of inflammation and oxidative stress in NAFLD, GSE affects the severity of hepatic steatosis by reducing inflammatory factors such as hs-CRP, TNF-α, and oxidative stress (MDA), as well as increasing antioxidant enzymes (SOD and CAT) [55, 56]. However, no significant effect on liver enzymes was observed in interventions made with grape products on patients with high blood pressure or obese individuals [57, 58]. The difference in intervention subjects can be one of the reasons for the differing results. Between-group analysis in this study showed a significant decrease in liver enzyme levels and the severity of hepatic steatosis in the GSE group compared to the control group. Similar findings have been observed in several studies showing the reductive effects of GSE on lipid metabolism. It is indicated that GSE can suppress lipogenesis and increase hepatic beta-oxidation by affecting the gene expression of hepatic lipid droplet proteins, sterol regulatory element-binding protein 1c (SREBP-1c), and peroxisome proliferator-activated receptor-α (PPAR-α) [59].

Among the strengths of the present study, we can mention the more accurate method of the present study and the evaluation of the variables such as Insulin levels, HOMA-IR, HOMA-β, QUICKi index, WC, HC, WC/HC, blood pressure variables and atherogenic index of plasma (AIP). Also, in the section of statistical methods, many confounding factors were considered, and the ITT method was used to compensate for the missing items. The study limitations include a small sample size and short duration, highlighting the need for future studies to address these constraints. Moreover, ultrasound’s limitation in distinguishing between steatosis and steatohepatitis without liver biopsy is acknowledged, despite its common use as an initial diagnostic tool for hepatic steatosis in suspected NAFLD patients. While ultrasound proves highly effective in detecting steatosis when over 33% of hepatocytes are affected, its reliability diminishes in cases of mild fatty infiltration [60].

## Conclusion

According to the results of the present study, it can be said that the supplementation of GSE for 2 months can be effective in improving the lipid profile, insulin resistance, blood pressure, and the severity of hepatic steatosis in patients with NAFLD. Therefore, the supplementation of GSE can be considered as a therapeutic solution to improve the symptoms of patients with NAFLD. Although more research is needed to validate these findings, our study does indicate some early positive impacts in patients with NAFLD.

## Data Availability

The data presented in this study are available on request from the corresponding author. The data are not publicly available due to privacy restrictions.

## Abbreviations

BMI: body mass index
WC: waist circumference
HC: hip circumference
FBS: fasting blood sugar
HbA1c: glycosylated hemoglobin
HOMO-IR: homeostasis model assessment of insulin resistance
QUICKi: quantitative insulin sensitivity check index
HOMA-β: homeostasis model assessment of β-cell function
TG: triglyceride
TC: total cholesterol
HDL-c: high-density lipoprotein cholesterol
LDL-c: low-density lipoprotein cholesterol
VLDL: very low density lipoprotein
ELISA: enzyme linked immunosorbent assay
